# An Increase of Plasma Advanced Oxidation Protein Products Levels Is Associated with Cardiovascular Risk in Incident Peritoneal Dialysis Patients: A Pilot Study

**DOI:** 10.1155/2015/219569

**Published:** 2015-10-25

**Authors:** Elena Gonzalez, Maria-Auxiliadora Bajo, Juan J. Carrero, Bengt Lindholm, Cristina Grande, Rafael Sánchez-Villanueva, Gloria Del Peso, Mariana Díaz-Almirón, Pedro Iglesias, Juan J. Díez, Rafael Selgas

**Affiliations:** ^1^Department of Nephrology, Hospital Universitario La Paz, IdiPAZ, Spain; ^2^Renal Medicine and Baxter Novum, Karolinska Institutet, Stockholm, Sweden; ^3^Department of Biochemistry, Hospital Universitario La Paz, IdiPAZ, Spain; ^4^Section of Biostatistics, Hospital Universitario La Paz, IdiPAZ, Spain; ^5^Department of Endocrinology, Hospital Universitario Ramón y Cajal, Spain

## Abstract

Advanced oxidation protein products (AOPPs) are considered as markers and even mediators of the proinflammatory effect of oxidative stress in uremia. We hypothesized that an increase of oxidative stress associated with peritoneal dialysis (PD), estimated by the variation of plasma AOPPs over time, might be associated with cardiovascular (CV) risk and overall prognosis. In 48 PD patients, blood samples were collected on two occasions: the first one in the first six months after starting PD therapy and the second one, one year after. The plasma AOPPs level variation over the first year on PD was significantly associated with CV antecedents and also with CV prognosis. In those patients in whom the AOPPs levels increased more than 50% above the baseline value, a significant association with past and future CV disease was confirmed. These patients had 4.7 times greater risk of suffering later CV disease than those with a smaller increase, even after adjusting for previous CV history. Our data suggest that the increase of AOPPs plasma level over the first year on PD is conditioned by CV antecedents but also independently predicts CV prognosis. AOPPs plasma levels seem to represent the CV status of PD patients with sufficient sensitivity to identify those with a clearly sustained higher CV risk.

## 1. Introduction

Cardiovascular disease (CVD) is the leading cause of morbidity and mortality in end-stage renal disease patients [1]. Besides the traditional risk factors for CV events such as hypertension, diabetes mellitus, and hyperlipidemia, “non-traditional” factors, such as oxidative stress, abdominal fat deposition, and endothelial dysfunction, have also been proposed [2, 3]. Oxidative stress, defined as the tissue damage resulting from an imbalance between an excessive generation of oxidant compounds and insufficient antioxidant defense mechanisms, probably contributes to endothelial dysfunction and atherosclerosis and, therefore, CV complications [4].

Witko-Sarsat et al. [5] found that, due to the oxidative damage, proteins can modify their spectroscopic characteristics. These altered proteins, named advanced oxidation protein products (AOPPs), have a molecular weight of 600 kDa and are highly elevated in hemodialysis (HD) patients. The same authors demonstrated that AOPPs act to trigger the oxidative burst and the synthesis of inflammatory cytokines in neutrophils and monocytes [6]. Since glycation-modified proteins also induced protein cross-linking and are elevated in uremic patients, AOPPS and advanced glycation end products (AGEs) are highly correlated [6].

Oxidative stress causes damage to important biological structures and may enhance the inflammatory response. New compounds, such as AOPPs, but also AGEs and advanced lipoperoxidation end products (ALEs) may constitute a new molecular basis for the deleterious activity of oxidants, and they could be considered to be true mediators of the proinflammatory effect of oxidative stress in uremia [7, 8].

Moreover, these authors have also evaluated the relationships between plasma AOPPs and markers of monocyte activation in uremia and demonstrated a high correlation between AOPPs and renal creatinine clearance and inflammatory cytokine levels such as tumor necrosis factor alpha (TNF-*α*) [5, 9–12].

Residual renal function (RRF) affects the survival rate and the development of CVD in peritoneal dialysis (PD) patients. In incident PD patients, lower RRF has also been associated with increased inflammation. Loss of RRF is associated with increased AOPP and AGEs plasma levels, suggesting that preservation of RRF has a beneficial effect on reducing the oxidative stress in PD patients [11].

Endothelial dysfunction is an early initiating event in atherosclerosis and a risk factor for future CV events. Oxidative stress and uremia-related CV risk factors probably play a role in the pathogenesis of endothelial dysfunction [11, 12]. In a previous article [13], our group demonstrated that peritoneal protein clearance (PrC) and 24 h effluent peritoneal protein losses (PPL) on initiating PD are directly and independently related to peripheral arterial disease (PAD), as an expression of the highest CV disease grade. A greater rate of peritoneal transported protein might be the result of peritoneal endothelial dysfunction, reflecting systemic endothelium damage.

We hypothesized that an increase of oxidative stress associated with PD and estimated by AOPPs plasma level variation over time might be associated with CV risk factors development and overall PD patient prognosis.

Therefore, our primary objective was to evaluate, in this pilot study, the association of dynamic plasma AOPPs levels with CV background and outcome in a cohort of incident PD patients. Our secondary objective was to explore the biological variation of AOPPs plasma levels throughout a year in this high risk population and to study the influence of renal and peritoneal functions on this variation.

## 2. Patients and Methods

### 2.1. Patients

We studied 48 patients who remained at least one year in the PD program of the Hospital Universitario La Paz, Madrid, Spain. Patients comprised 37 men and 11 women. Their mean age was 54.0 ± 15.9 years and at baseline mean duration of preceding time on PD at inclusion was 6 months. There were 11 (22.9%) patients with diabetes, 42 (87.5%) with hypertension, and 27 (56.3%) with previous CVD. Plasma samples were obtained from each patient on two occasions: the first one in the first six months after starting the PD therapy (period between 2000 and 2009) and the second one, one year after. The following information was collected from patient records: demographic data (including age, sex, height, weight, and body mass index); prevalence of CV risk factors (hypertension, diabetes mellitus, hyperlipidemia, CV disease at the beginning of dialysis, smoking habits, and secondary hyperparathyroidism); laboratory tests (blood glucose levels, serum levels of cholesterol, albumin, triglycerides, albumin, and high-sensitivity C-reactive protein (hs-CRP)); and PD-related parameters: type of dialysis (continuous peritoneal ambulatory dialysis (CAPD) or automated peritoneal dialysis (APD)), RRF, and urea (U-MTAC) and creatinine (Cr-MTAC) mass transfer area coefficients. All patients gave their consent to give blood samples in order to participate in the study.

Patients were followed up until death or end of follow-up (January, 2014). Causes of deaths and CV events were determined by clinicians based on clinical presentation and examination of patients. CV events included electrocardiographically documented angina, myocardial infarction, heart failure, atrial fibrillation, stroke, and peripheral vascular disease.

### 2.2. Methods

AOPPs classic determination was based on spectrophotometric detection according to Witko-Sarsat et al. [5]. In order to minimize the impact of storage time and the influence of triglyceride, we decided to use the modified AOPP assay developed by Anderstam et al. [14]. The modified AOPP assay included, in addition to the Witko original AOPP methodology, a sample preparation procedure to precipitate lipoproteins (very low density lipoproteins (VLDL) and low density lipoprotein (LDL)) in the plasma (Konelab HDL-cholesterol precipitating reagent, Thermo Electron Corporation, Vantaa, Finland). This reagent is normally used as a preparation step before determination of HDL-cholesterol on Konelab analyzers. Fifty *μ*L of reconstituted precipitating reagent (dextran sulphate and magnesium ions) was mixed with 500 *μ*L of EDTA plasma, centrifuged at 1000 ×g for 20 min, upon which the supernatant was carefully removed. AOPPs were immediately measured in the supernatant at 340 nm on a microplate spectrophotometer under acidic conditions and expressed as chloramine-T equivalents (*μ*mol/L). Glomerular filtration rate (GFR) was expressed calculating the mean between creatinine and urea kidney clearances. Peritoneal protein clearance was assumed to be peritoneal albumin clearance as almost all protein in peritoneal effluent is albumin (the plasma measurement for the calculation was albumin).

All patients were subjected to a baseline peritoneal kinetic study (within 4 weeks after the start of dialysis) and one year after. This study was performed using a standard protocol of four-hour dwell period with 3.86% glucose concentration 2 L volume exchange. During the peritoneal function study, the patients fasted and were given no medication except for low doses of subcutaneous insulin as necessary. To measure the diffusive capacity, six samples of the peritoneal effluent were collected (at time 0, 30, 60, 120, 180, and 240 minutes) and a blood sample was also taken. Based on these determinations, D/P Cr was calculated as described by Twardoski et al. [15], and mass transfer coefficients of urea (urea-MTAC) and creatinine (cr-MTAC) were calculated based on a mathematical model described previously by our group [16].

### 2.3. Statistical Analysis

Results are expressed as mean ± standard deviation (SD) for normally distributed continuous variables, or median (interquartile (IQ) range) for nonnormal data, or percentage of total, as appropriate, for categorical variables. Participant data were compared by using a chi-square test, Fisher test, Student's *t*-test, Wilcoxon signed-rank test, or Mann-Whitney *U*-test, as appropriate. Spearman correlation analysis was used to examine the significance of associations between variables. Two-tailed 95% confidence intervals (CI) and *p* values are presented with *p* < 0.05 regarded as significant. All statistical analyses were performed using statistical software SPSS for Windows, version 15.0 (Chicago, SPSS Inc., USA).

## 3. Results

We studied 37 men and 11 women. Mean age was 54.0 ± 15.9 years and mean duration of preceding time on PD at inclusion was 6 months. There were 11 (22.9%) patients with diabetes, 42 (87.5%) with hypertension, and 27 (56.3%) with previous CVD. The main clinical, analytical data and kidney and PD function parameters throughout the study period are reported in Table 1.

In univariate analysis, plasma AOPPS levels were neither associated with demographic, clinical, or kidney variables nor associated with peritoneal function parameters. Baseline AOPPs levels were positively correlated (Spearman Rho 0.69, *p* < 0.01) with AOPPs levels at 1 year.

Mean time of follow-up after the second AOPPs determination was 71.4 ± 38 months (median 67 [5–151 months]). During that period, 28 patients (58.3%) had undergone renal transplantation, 9 (18.8%) had been transferred to hemodialysis, and 9 (18.8%) had died during PD therapy. Thirteen patients died during the total follow-up (*census date*: January, 2014); CV disease was the most common cause (7 patients), followed by infection (4 patients). In the univariate Cox proportional hazards model, age, presence of diabetes or CV disease, serum albumin concentration, CRP level, PD modality, peritoneal parameters, or residual renal function was not associated with mortality.

**Figure 1:**
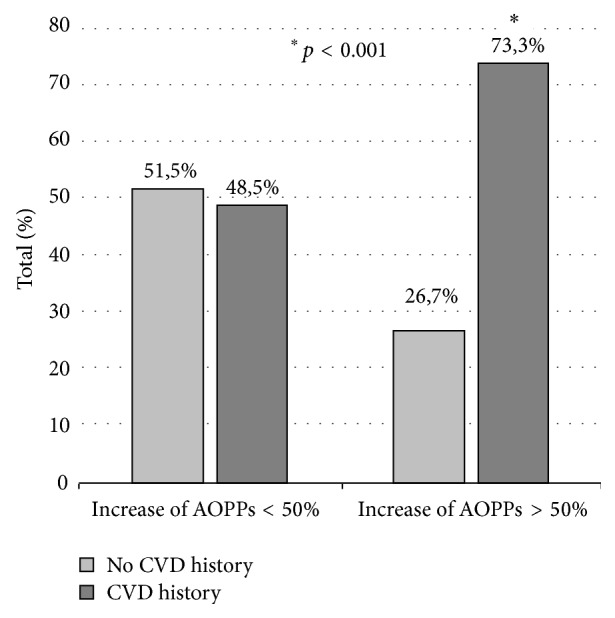
Prevalence of cardiovascular disease (CVD) according to the change of plasma AOPPs levels after one year on PD. Among patients with an increase lower than 50% (*n* = 33), there was no difference in prior CV disease prevalence whereas, among those with AOPPs increase higher than 50% (*n* = 15), there was a higher prevalence of prior CV disease. ^*∗*^ *p* < 0.001 (significant CVD history in patients with an increase of plasma AOPPs levels greater than 50% versus lower than 50%).

Since there were individuals who increased their AOPPS plasma levels during the study period while others decreased them, we assumed that the analysis using the median value would not differentiate differences among patients. For this reason, and due to the lack of literature to recommend a clear cut-off value for the analysis, we decided to compare the groups according to the increase or decrease of the baseline AOPPs level and its magnitude (percentage) of change (median value 29.6%, with a range from −63.3 to +998.4%). Forty patients (83.3%) showed an increase of plasma AOPPs level at one year (in 14 of them this increase was > 50% from baseline value). The AOPPS levels decreased only in 8 (16.7%) patients. In those patients in whom the AOPPS levels increased more than 50% of baseline value, an association with past and future CV disease was found. A direct relationship between the percentage increment in AOPPs level at 12 months and CV antecedents was found (effect size; phi = 0.605, *p* < 0.001). In fact, patients with a CV history had 8.4 times higher risk (95% CI [2.09, 33.48]) to present a percentage of AOPPs increase greater of 50% at month 12 of PD treatment than those patients with no CV disease (Figure 1).

**Figure 2:**
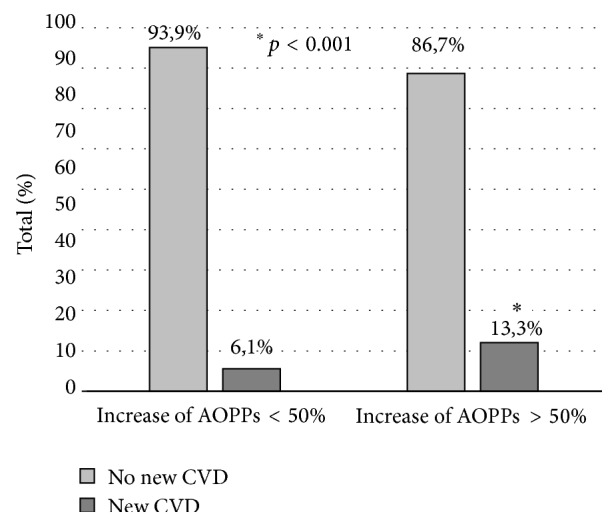
Incidence of new cardiovascular (CV) disease according to changes of plasma AOPPs level during one year on PD. Patients with an increase of AOPP higher than 50% (*n* = 15) had 4.7 times greater risk of developing a new CV event than those with a smaller increase of AOPP (*n* = 33). ^*∗*^ *p* < 0.001 (significant CV disease in patients with an increase of plasma AOPPs levels greater than 50% versus lower than 50%).

The percentage of increase in AOPPs at month 12 was also significantly associated with the development of new CV disease (effect size; phi = 0.612, *p* < 0.001). Among the patients who developed a CV event, the percentage of patients showing an increase of AOPPs greater than 50% at month 12 was significantly higher than those patients with an increase lower than 50% (64.3% versus 7.7%, *p* < 0.01). The first group of patients had 4.7 times greater risk (95% CI [2.04, 11.05]) to suffer later CV disease than those with the smaller increase (Figure 2), even after adjustment for prior CV disease history (we first ruled out that the AOPPs levels were a modifier factor between the risk to develop a new CV event in patients with a CV history (Breslow-Day test, *p* = 0.23) and also that it was a confusion factor (Mantel-Haenszel test, *p* < 0.05)).

## 4. Discussion

Our interest has focused on the estimation of the oxidative stress of PD patients by measuring circulating AOPPs over a period of time on risk and to relate their behavior to CV status. The main finding of our study was that plasma AOPPs levels increase over time mostly among patients with CV history and also in patients with subsequent CV events suggesting that the change in AOPPs level represents a marker of a permanent CV risk status.

The median AOPP value in our study is in agreement with previous literature [17, 18], which uses the same methodology. To our knowledge, ours is the first study in which the AOPPs values were measured in the same patients at two different time points. Alike other markers such as CRP, it seems that AOPPs could be more valuable when being periodically monitored in clinic rather than when assessed as an isolated value. A prooxidant status defined by continuous increase in AOPPs levels would thus reflect a CV prone-event status. Probably, the prooxidant status will maintain a permanent endothelial dysfunction and promote new CV events. The opportunity of reducing this process and to estimate this reduction by AOPPs plasma levels give to our data potential clinical usefulness.

Dialysis patients have an increased risk of CV morbidity and mortality. Nonclassical CV risk factors such as inflammation, malnutrition, endothelial dysfunction, and oxidative stress have been suggested to be responsible for this risk [1, 2]. Witko-Sarsat et al. [5] first described the presence of high levels of plasma oxidized proteins in hemodialysed patients and named them AOPPs. They found that these altered proteins seem to act not only as true inflammatory mediators, but also as uremic toxins with proinflammatory effects [6]. Besides, CKD is a low grade inflammatory process due to several mechanisms such as a failure of reactive oxygen species (ROS) clearance or a low level of antioxidant vitamins due to dietary restrictions (fruits and vegetables).

Oxidative stress is also the unifying mechanism for many CV risk factors, which additionally supports its central role in CV disease. The majority of CV disease results from complications of atherosclerosis [19–22]. An important initiating event for atherosclerosis may be the transport of oxidized-LDL (Ox-LDL) across the endothelium into the artery wall. Endothelial cells, smooth muscle cells, and macrophages are the sources of oxidants in the cells, which induce the expression of adhesion molecules and chemotactic factors. These processes lead to the activation, attachment of T lymphocytes and monocytes to the endothelial cells, and the generation of reactive oxygen species (ROS), which convert Ox-LDL into highly oxidized LDL, which, in turn, will form foam cells.

Although the concept of atherosclerosis as an inflammatory disease is now well established, evidence suggests that chronic inflammation may be considered a common pathogenic step in the pathogenesis of insulin resistance, diabetes, atherosclerosis, and CV disease [23]. Inflammation is one manifestation of oxidative stress, caused by a mitochondrial overgeneration of free radicals, and the pathways that generate the mediators of inflammation, such as adhesion molecules and interleukins, are all induced by oxidative stress.

Our data did not show a relationship between plasma AOPPs and RRF, which disagrees with previous studies performed on PD patients [24]; although our small sample size could mask a real association, it may also be related to the greater RRF and diuresis of our patients as compared to previous literature, even at one year after starting PD [24]. The suggestion of AOPPs as an endogenous nephrotoxic agent cannot be confirmed by our data.

However, to our knowledge, this is the first study analyzing the relationship between AOPPs and CV disease in PD patients. The difficulty in performing the AOPPs determination encourages conducting a pilot study before performing larger studies or introducing them into clinical practice. Due to the relatively low number of patients in this study, the results should be interpreted with caution; on the other hand, we were able to identify statistically significant associations related to AOPPs levels. Nevertheless, the small sample size and the difficulty of measuring these markers are the main limitations of our study. Furthermore, we cannot dismiss the possibility that RRF may have interfered with the results. Nevertheless, the possibility to include AOPPs as a new marker to predict or measure “non-classical” CV risk in patients at high risk, such as those on PD, should be considered when designing further clinical studies to confirm and further exploit these results.

In conclusion, the results of the current pilot study suggest that the increment of plasma AOPPs levels over the first year on PD associates with CV antecedents and also relates to the risk of developing new CV events. The plasma AOPP levels may be a useful marker that can represent the CV status of PD patients with sensitivity to reflect changes in those patients at a clear higher permanent CV risk.

## Figures and Tables

**Table 1:** Clinical and biochemistry values and kidney and peritoneal function parameters in 48 PD patients at inclusion and at one year after starting PD.

	PD patients (*n* = 48)	
	Baseline	One year after
BMI, kg/m^2^	25 ± 3.3	25.4 ± 3.2
Cholesterol, mg/dL	175 (149–199)	164 (145–187)
Triglycerides, mg/dL	121 (97–188)	107 (85–123)
Albumin, g/dL	3.7 ± 0.4	3.7 ± 0.4
hs-CRP, mg/L	1.5 (0.7–4.7)	1.9 (0.7–3.2)
AOPPs, *μ*mol/L	76.6 (61.4–92.3)	95.2 (75.3–126.3)

Residual diuresis, mL/24 h	1713 ± 1125	1302 ± 945
Residual GFR, mL/min/1.73 m^2^	5.9 ± 3.6	4.3 ± 3.4
Creatinine MTAC, mL/min	8.7 ± 2.7	8.7 ± 4.3
Urea MTAC, mL/min	22.4 ± 4.5	22 ± 6.9
Ultrafiltration, mL/4 h (dwell time 3.86% glucose)	708 ± 241	693 ± 265
Peritoneal protein clearance, mL/day	4.7 ± 3.5	4.9 ± 3.8

Values are presented as mean ± SD for normally distributed variables, or median (interquartile range) for nonnormal data.

BMI: body mass index; hs-CRP: high-sensitivity C-reactive protein; AOPPs: advanced oxidative protein products; GFR: glomerular filtration rate; MTAC: mass transport area coefficient.
